# The Treatment of Chronic Complex Regional Pain Syndrome with Novel Neuromodulatory Sound Waves: A Case Report

**DOI:** 10.3390/healthcare12161640

**Published:** 2024-08-17

**Authors:** Lee Bartel, Peter Dyback, Aslam Khan

**Affiliations:** 1 Faculty of Music, University of Toronto, Toronto, ON M5S 2C5, Canada; 2 Neuro Spinal Innovation Inc., Mississauga, ON L5L 3K3, Canada

**Keywords:** complex regional pain syndrome, CRPS, neuromodulation, pulsed stimulation, vibration, chronic pain, sound stimulation

## Abstract

This paper presents a case of a 35-year-old female patient diagnosed with Complex Regional Pain Syndrome (CRPS) type I and treated over a two-month period with a novel low-frequency sound-transduced focal pulsed stimulus. The patient received 21 treatments consisting of focally applied sound sweeps in the 15–100 Hz range. Outcome measures included the Visual Analogue Scale for pain, five physical assessment parameters, medication, and the Pain Catastrophizing Scale. A follow-up was conducted at six months. The results show that the patient’s low-back pain level was substantially reduced after treatment and after six months. CRPS-related peripheral pain was strongly reduced but had some rebound after six months. The low-frequency sound-transduced focal pulsed stimulus shows potential as a non-invasive treatment for CRPS and deserves controlled clinical trials.

## 1. Introduction

Complex Regional Pain Syndrome (CRPS) is a persistent, intense, chronic pain condition typically involving the limbs and extremities for which the cause is not yet fully understood [1]. A common view is that CRPS results from central and peripheral nervous system dysfunction including the brain and spinal cord [2]. Two types of CRPS are diagnosed: type I without evident nerve damage and type II when there is known nerve damage. Although the pathogenesis is not yet clear, research has implicated neuropathic inflammation, especially of peripheral nociceptors, and an imbalance in the autonomic nervous system [1].

Since 2003, CRPS has been diagnosed with the Budapest criteria [3], which include the following: (1) on-going pain out of proportion with any possible incident, (2) patient self-report of a symptom in at least three of the categories sensory (e.g., extreme pain with a gentle touch), vasomotor (e.g., hot flush and changed skin color), sudomotor/edema (e.g., sweating or swelling), or motor/trophic (e.g., reduced range of motion), and (3) the patient’s condition has no other diagnosis that better explains it [3]. In addition to pain, patients with CRPS commonly experience depression, anxiety, diminished function, and poor quality of life. In addition, research shows that CRPS is associated with neuropsychological symptoms such as brain fog, memory access, word retrieval, and sleep deficits; physical symptoms such as weakness and lethargy; and gastrointestinal symptoms such as nausea, indigestion, and diarrhea/constipation [4].

The epidemiology of CRPS is generally not well established, with considerable differences reported among studies. For type I in adults, numbers vary from 26.2 cases per 100,000 person-years to 5.46 per 100,000 person-years [4]. In the pediatric population, there are less data, but an incidence of 1.2 per 100,000 is estimated [5]. Occurrence is generally higher among females than males.

Treatment for CRPS is most effective when started early, but since onset may be in stages and the pain is not immediately diagnosed as CRPS, this is often not the case. The most common treatments first prescribed and considered most important are physical and occupational therapies [1,6], but the inclusion of pharmacological, psychiatric, and patient education are also important. Although no conservative treatment has been found to be fully rehabilitative for chronic CRPS, many are tried including acupuncture, biofeedback, contrast baths, counter strain, electrotherapy including spinal cord and implanted neural stimulation, gentle range of motion training, isometric strengthening exercises, and massages. Some research points to graded motor imagery and mirror therapy as being of greatest rehabilitative benefit but the quality of evidence for this is very low [1]. Formerly used treatments but rarely used now include sympathetic nerve blocks, surgical sympathectomy, and even amputating the painful limb. Since no medications are specifically recommended for CRPS by the U.S. Food and Drug Administration (FDA), physicians recommend a large range including basic acetaminophen and non-steroidal anti-inflammatory drugs (NSAIDS), neuropathic drugs such as gabapentin and pregabalin, topical anesthetic ointments such as lidocaine and patches such as fentanyl, biophosphonates, corticosteroids, botulinum toxin injections, opioids, and controversial treatments such as N-methyl-D-aspartate (NMDA) receptor antagonists like ketamine [6].

A dimension of CRPS and chronic central pain in general that may not receive adequate attention is a neurogenic component—thalamocortical dysrhythmia (TCD) [7]. Studies that have examined brain oscillation patterns related to chronic pain and CRPS specifically have shown the presence of TCD [8,9,10]. Also associated with neurogenic pain is neuroinflammation, seen in both CRPS and fibromyalgia [11]. A treatment modality used with some success to re-regulate brain oscillatory dysrhythmia and chronic pain is electrical stimulation [12,13,14]. Non-electrical neuromodulation techniques including visual, auditory, and vibrotactile flickers or vibrations also appear to be effective in oscillatory modulation and inflammation reduction [15,16,17]. Indeed, relatively short low-frequency auditory sweeps have been shown to reset the 40 Hz gamma activity implicated in TCD [18].

The treatment used in this case of CRPS was a focally applied low-frequency sound sweep transduced into a focal pulsed stimulus in the 15–100 Hz range, best described as **a**ccelerated audible **l**ow-frequency **kin**etically **d**irected **i**mpulses (ALKINDIs), meaning the following: “accelerated”—a rapidly increasing frequency curve, “audible low-frequency”—at the lowest level of human audibility in the 15–100 Hz range, “kinetically directed”—very specifically angled and directed at body targets, and “impulses”—sound-initiated percussion waves. This stimulus has been shown to be effective when applied to vertebrae for spine-related pain [19]. But since back pain has a neurological dimension [20], and the sound stimulus has also been shown to have an mRNA effect [21], it was used experimentally in this case as a neuromodulatory stimulus directly on the skull.

## 2. Case Presentation

The patient was a 35-year-old woman who presented to a Neuro Spinal Innovation (NSI) clinic in Mississauga, Ontario, with severe bilateral chronic pain in both legs from her knee down, with stiffness in her joints and muscles spasms. She described these spasms as cramps similar to “Charlie horses” in her calf and foot, primarily in her right leg. She also described occasional acute pain in her hips. She reported a condition in her arms similar to her legs accompanied by shaking. In general, she was experiencing extreme fatigue and weakness. Her history revealed that prior to the development of severe pain, she was very athletic, playing sports and enjoying varied physical activity, and was very socially engaged with friends. The pain condition developed over some years and had persisted for many. She first developed Temporomandibular Joint (TMJ) pain and low-back and shoulder pain in 2000. In 2005, she developed hip and leg pain, with particular pain in her knee and ankle. In 2005, she received three spinal nerve blocks and three epidural steroid spinal injections. In 2006, she was diagnosed with Reflex Sympathetic Dystrophy (RSD, a previous name for CRPS) and prescribed a wheelchair and used a cane to walk. Then, in 2007, her shoulder pain extended to arm, elbow, and wrist pain.

In 2012, the patient was assessed at the RSD/CRPS Treatment and Research Institute in Tampa, Florida. At that time, she described her condition as follows: “Chronic pain and swelling. Both feet and hands swell and pain is from both hips down to my toes and shoulder to fingers. Reaching causes pain in my elbows. Walking hurts my feet and knees. It’s hard to keep going. Sitting for extended periods makes my feet swell and hurt. Kneeling hurts my knees along with walking up stairs. Memory is affected—I forget things easily. It’s hard to complete tasks. My brain feels foggy—it affects my concentration and understanding what someone’s saying to me. Hard to process instructions. My hands ache and shake. It’s hard to write and I drop things often. I feel isolated from others because no one understands”. She described in some detail how her personal care, meals, house and yard work, getting around, shopping, interests, and social activities were all negatively affected, how this disability made her feel guilty that she could not do more, how it isolated her because of the pain and embarrassment, and how it resulted in extreme anxiety, depression, and occasional panic attacks. After a case review and physical examination, including the determination of pain thresholds, the patient was diagnosed with Complex Regional Pain Syndrome (CRPS) type I. The doctor at that point recommended a four-day course of ketamine treatment, which the patient refused. She was taking pregabalin.

Five months after the diagnosis, the patient was admitted to a hospital as a result of a suicide attempt. On the hospital safety plan, she listed the following as symptoms: “isolation, feeling numb with no emotions, helplessness, feeling I am a burden on others, feeling a sense of emptiness”. On a more detailed form, she indicated her three biggest fears as follows: “The chronic pain from the illness will continue to get worse, I won’t be able to have a child, and I’ll die young”.

From 2012–2022, the patient continued to suffer with this debilitating condition, spending much of the last two years in bed. During this time, she tried acupuncture, massage therapy, chiropractic, and naturopathic medicine, with little relief.

At the time of presentation at the NSI clinic in 2022, the case met the Budapest criteria, as described above and as reported in the physical examination below. In addition, she was taking gabapentin twice daily (total 800 mg), Buprenorphine (24 mg) daily, and Xanax (2 mg) less than once a day. The patient voluntarily supplied medical records and has given informed written consent for her clinical information to be reported in this study.

### 2.1. Physical Examination and Diagnosis

Part of the diagnosis used by the NSI clinic is a series of physical and neurological tests, reported in Table 1 as the five pre-treatment assessment parameters. The five parameters include the following: (1) the degree of tilt of the shoulder and pelvis in the coronal plane (measured with a set of calipers to a quarter degree); (2) supine leg length discrepancy (LLD); (3) coordination through response to resistance of the arm and leg; (4) range of motion of the cervical spine (ROM); and (5) tender spots associated with deep spinal palpation. These five diagnostics are routinely performed before and after each treatment and indicate the status of the physical and neurological condition at the time, allowing us to track any change in a course of treatment. These five assessment parameters (FAPs) are an established part of the NSI treatment for spine-related pain. Since CRPS is regarded both as a neurological and spinal condition, it was considered appropriate to use these parameters in this experimental case as well.

At the initial examination, the patient presented with (1) a shoulder tilt deviation to the right by 1.5 degrees and a pelvic tilt deviation to the right by 2 degrees; (2) a supine leg length 0.5 cm shorter on the right side compared to the left side; (3) an arm coordination response to resistance of 4/5 on the left and 3/5 on the right; (4) a leg coordination response to resistance of 4/5 on the left and 3/5 on the right; (5) a cervical range of motion (ROM) mildly affected on both sides; and (6) painful tender lesions (29 severe (red) and 11 moderate (yellow)) found along the spine.

At the time of admission to the NSI clinic, the patient rated her pain on the Visual Analogue Scale (VAS) at multiple locations out of a total of 10, where 10 is the worst pain possible (rounded to the nearest quartile). Her pain ratings showed that her legs were most affected, with a range of rating from 10 to 8.25 for her legs. Her arms and shoulders were painful but somewhat less so, with a range from 5.5 to 3.75. Her low back was also a location of substantial pain, with a rating of 7.5 (See Table 2 for specific location ratings.)

A common effect of chronic pain, especially following a traumatic incident, is the psychological catastrophizing of the situation to the point of significantly contributing to the debilitating effects. The Pain Catastrophizing Scale (PCS) [22] was used to assess the degree of catastrophizing in this case. It was administered before the treatment and then again in a six-month follow-up.

A review of the case history reported, the pain location ratings, the patient’s experience description, and the data from the physical examination confirmed the previous diagnosis of Chronic Regional Pain Syndrome.

### 2.2. Treatment Plan

**Figure 1:**
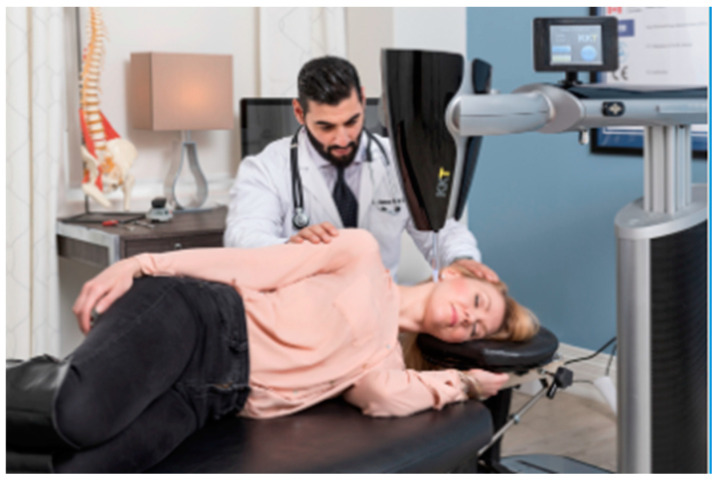
The NSI treatment device. Note: This is not the clinician or patient reported here. The image is owned and provided for use with permission by NSI.

The treatment device used in this study is a sound transducer-driven, vibratory stylus-mediated, impulse delivery mechanism. The device head is mounted on a flexibly positioned armature on a stand [19]. The head of the device, moveable in three dimensions, is positioned by the clinician and then fixed in location at the prescribed location and angle. The stylus is pressure-sensitive and so provides patient safety by being collapsible if the patient moves out of position (Figure 1) [19]. The NSI device’s sound transducer generates waveforms best described as **a**ccelerated audible **l**ow-frequency **kin**etically **d**irected **i**mpulses (ALKINDIs), meaning the following: “accelerated”—a rapidly increasing frequency curve, “audible low-frequency”—at the lowest level of human audibility in the 15–100 Hz range, “kinetically directed”—very specifically angled and directed at body targets, and “impulses”—sound-initiated percussion waves. ALKINDIs are focused through a stylus onto particular locations on the spine, skull, or body. At the initial assessment, treatment parameters were determined from digital data captured through X-rays of the spine, although currently 3D photo imaging can be used instead. Table 3 indicates the anatomical locations at which the treatment was applied in this case and the number of pulses used during each session.

Because CRPS is considered a disorder of the brain and spine, the anticipated treatment plan was to apply the ALKINDIs to the spine (particularly C1) to stimulate changes in alignment and to the skull at the top center, known in the 10–20 system of electroencephalography (EEG) as Cz, in order to drive a neuromodulatory effect. The number of treatments was to be determined by response stability, while frequency was planned to be three or four times a week, as this was possible according to patient availability. The ALKINDI treatment is most commonly used for spine-related pain and typically follows a pattern of three treatments per week for the first 4 weeks, with the most structural and pain relief progress observed in the first 2 weeks and then stabilization in the next 2 weeks. Typically, if the pre-treatment parameters are observed as stable, around the 12th treatment, patients are transitioned into a maintenance phase, with treatment once a week for another six treatments. In this case, the treatment was used in an experimental manner, and so, treatment frequency was more case-specific, with anticipation of neuromodulation as a dominant factor. Also, the patient’s home was 2 h from the clinic and so availability was a factor determining frequency. Note that the 12th treatment was on a weekly basis due to availability but returned to the more frequent basis in week 5.

Focal treatment location decisions were made in this case partially on the premise that CRPS has both a neurological dimension treated with Cz and a spine-related dimension treated primarily with C1. However, the clinician made some decisions based on the pre-treatment parameters reported in Table 1 and based on the patient’s self-report. The actual treatment delivered and specific focal location are reported in Table 3.

### 2.3. Outcomes

The ALKINDI stimulating treatment has in previous studies been shown to have a physiological orthopedic effect, as measured by the five assessment parameters [19,23,24,25]. In this case, a rapid improvement was seen in most of the parameters (Table 1) within the first week of treatment, and by the end of the second week of treatment, all five parameters were neutral, although complete stability was not fully established by the end of treatment. For example, range of motion (ROM) was still mildly affected at the seventh treatment but remained normal from that point on. The number of tender spots from palpation initially was high, with 29 severe and 11 moderate spots, and by the seventh treatment was zero, with only low numbers showing periodically from that point.

A planned reassessment of pain with the VAS was conducted before the third and eight treatments. The results, reported in Table 2, showed that by the end of the second week of treatment (eighth treatment), the average pain level had been reduced by 80.3% from the initial assessment.

A six-month follow-up was performed with the patient who then reported pain levels higher than when the treatment ended but substantially lower than before the treatment began (see Table 2). Her activities of daily living were reported as approaching normal—social activities with friends, participating in household activities, working on her job resume, etc. An informal follow-up at 2 years after the end of the treatment revealed that she is working full-time and is engaged to be married.

Despite the levels of pain reported and the length of time during which the condition was experienced, the PCS score obtained before the treatment began was only 17/52, with the “Rumination” sub-score being 4, the “Magnification” sub-score being 5, and the Helplessness sub-score being 8. Six months after the treatment was ended, the PCS was again administered and resulted in a significantly higher score of 34/52, with the “Rumination” sub-score being 10, the “Magnification” sub-score being 6, and the Helplessness sub-score being 18. The noteworthy changes are the more than doubled “Rumination” and “Helplessness” sub-scores. One possible explanation for this is that before the treatment, the patient’s hope was high for possible relief, and given that although six months later the pain was substantially less than before the treatment, it was more severe than immediately following the end of the treatment. In this context of retrospection, the patient was not making the pain worse in her mind but the level of reflection on it and the sense of helplessness to defeat the pain may have resulted in higher catastrophizing scores. The patient was referred to a de-catastrophizing program called the Progressive Goal Attainment Program (pgapworks.com) run by Dr. Michael Sullivan, originator of the PCS.

In the pre-assessment, specific symptoms of CRPS other than pain were not examined in detail, with the clinician relying on previous formal assessments supplied and the patient’s self-report. In the six-month follow-up, the patient was asked to rate retrospectively example symptoms of sensory, vasomotor, sudomotor/edema, and motor/trophic conditions. The comparison was symptoms “in the past” with “present”. The results are reported in Table 4.

Following the treatments, the patient reported having reduced gabapentin from 800 mg to 400 mg daily, Buprenorphine from 24 mg daily to 8 mg, and Xanax to only occasionally.

## 3. Discussion

This case of long-standing chronic CRPS demonstrates the positive effects of focused low-frequency pulsed sound stimulation applied to the spine and to the skull. The ALKINDI treatment known in many parts of the world as the Khan Kinetic Treatment (KKT) has received regulatory approval from many regions including the FDA, Health Canada, and *Conformité Européenne* (CE) and is now also being used under the trade name SONIK, now with a portable hand-held device. This focused pulsed sound stimulation has been primarily used for spine-related pain conditions but with the assumption that the treatment not only results in spinal realignment but that the neurological dimension of back pain probably responds to the neuromodulatory effects of the gamma range pulsed stimulus, driving a brain response that affects circuit function and possibly regulation. Conditions such as CRPS that have a strong central nervous system component and brain circuit dysregulation [8,9,10,18,26,27] should then potentially respond to the neuromodulatory effect of the ALKINDI treatment. This case was conducted to explore this possibility and the results in this case strongly support this hypothesis.

Because this case demonstrated two possibly differentiated types of pain, low-back pain and chronic neurological extremity pain, separating those results may be instructive. The KKT/SONIK treatment has been used with back pain and the results have been demonstrated in multiple studies [19,24,25,28]. In this case, the patient rated her low-back pain at 7.5/10 before the treatment began, and by the eighth treatment, she rated it at 0.5. Most notably, at the six-month follow-up, she rated her low-back pain only at 1.0, demonstrating the long-term effect on spinal pain of the ALKINDI treatment. It should be noted that the treatment of C1 and lumbar and sacrum locations was directed at the low-back pain. The Cz location treatment was applied 11 times out of 21 treatments and was intended as gamma neuromodulation to reset and re-regulate brain circuits including the thalamocortical circuits. The pain reduction was rapid and dramatic, with an average of 80.3 percent reduction in peripheral pain. However, the long-term effect was less satisfactory, with the six-month follow-up showing pain returning to the 5/10 level. The implication may be that the neurogenic brain oscillation-based pain experience requires longer term treatment to fully rehabilitate the brain functions, or at least periodic maintenance treatments to sustain the effects.

## 4. Conclusions

This case of a young woman diagnosed with CRPS who experienced severe chronic pain for some 20 years demonstrates the potential efficacy of the ALKINDI treatment. Research is needed to explore the treatment protocols for this, the population for whom this might be most beneficial, and the more generalized efficacy with a larger population. A series of random clinical trials with rigorous controls is warranted for this. From a mechanism perspective, research is needed to explore the neuromodulatory effects on both the “connectome” and the “dynome” [29].

## Figures and Tables

**Table 1:** Results of five assessment parameters before treatments.

Tx	Shoulder/Pelvis	LLD	Coordination	ROM	Tender Spots
1 Wk1-T	S-Rt 1.5/P-Rt 2	Rt 0.5	Upper Lt 4 Rt 3	Lt mild	29 red
			Lower Lt 4 Rt 3	Rt mild	11 yellow
2 Wk1-W	S-Neutral/P-neutral	neutral	Upper Lt 5 Rt 5	Lt normal	1 red
			Lower Lt 5 Rt 5	Rt normal	7 yellow
3 Wk1-Th	S-Neutral/P-neutral	neutral	Upper Lt 5 Rt 5	Lt normal	0 red
			Lower Lt 5 Rt 5	Rt normal	8 yellow
4 Wk1-F	S-Rt 1/P-neutral	neutral	Upper Lt 5 Rt 5	Lt mild	5 red
			Lower Lt 5 Rt 5	Rt normal	8 yellow
5 Wk2-Su	S-Rt 1/P-neutral	Rt 0.5	Upper Lt 5 Rt 5	Lt normal	2 red
			Lower Lt 5 Rt 5	Rt normal	3 yellow
6 Wk2-Th	S-Neutral/P-neutral	Rt 0.25	Upper Lt 4 Rt 3	Lt mild	12 red
			Lower Lt 4 Rt 3	Rt mild	0 yellow
7 Wk2-F	S-Neutral/P-neutral	neutral	Upper Lt 5 Rt 5	Lt normal	0 red
			Lower Lt 5 Rt 5	Rt normal	0 yellow
8 Wk2-Sa	S-Rt 1/P-neutral	neutral	Upper Lt 5 Rt 5	Lt normal	0 red
			Lower Lt 5 Rt 5	Rt normal	0 yellow
9 Wk3-W	S-Neutral/P-neutral	neutral	Upper Lt 5 Rt 5	Lt normal	0 red
			Lower Lt 5 Rt 5	Rt normal	0 yellow
10 Wk3-Th	S-Neutral/P-neutral	neutral	Upper Lt 5 Rt 5	Lt normal	5 red
			Lower Lt 5 Rt 5	Rt normal	2 yellow
11 Wk3-F	S-Neutral/P-neutral	neutral	Upper Lt 5 Rt 5	Lt normal	0 red
			Lower Lt 5 Rt 5	Rt normal	0 yellow
12 Wk4-F	S-Rt 2/P-Rt 2	Rt 0.25	Upper Lt 3 Rt 3	Lt normal	0 red
			Lower Lt 3 Rt 3	Rt normal	0 yellow
13 Wk5-M	S-Neutral/P-neutral	neutral	Upper Lt 5 Rt 5	Lt normal	0 red
			Lower Lt 5 Rt 5	Rt normal	4 yellow
14 Wk5-T	S-Rt 2/P-Rt 1	Rt 0.25	Upper Lt 5 Rt 5	Lt normal	2 red
			Lower Lt 5 Rt 3	Rt normal	5 yellow
15 Wk5-W	S-Neutral/P-neutral	neutral	Upper Lt 5 Rt 5	Lt normal	1 red
			Lower Lt 5 Rt 5	Rt normal	3 yellow
16 Wk5-Th	S-Neutral/P-neutral	neutral	Upper Lt 5 Rt 5	Lt normal	0 red
			Lower Lt 5 Rt 5	Rt normal	0 yellow
17 Wk5-F	S-Neutral/P-neutral	neutral	Upper Lt 5 Rt 5	Lt normal	0 red
			Lower Lt 5 Rt 5	Rt normal	0 yellow
18 Wk7-M	S-Rt 0.5/P-Rt 1	neutral	Upper Lt 5 Rt 5	Lt normal	0 red
			Lower Lt 5 Rt 5	Rt normal	0 yellow
19 Wk8-T	S-Rt 1/P-neutral	neutral	Upper Lt 5 Rt 5	Lt normal	0 red
			Lower Lt 5 Rt 5	Rt normal	0 yellow
20 Wk8-W	S-Neutral/P-neutral	neutral	Upper Lt 5 Rt 5	Lt normal	0 red
			Lower Lt 5 Rt 5	Rt normal	0 yellow
21 Wk8-F	S-Neutral/P-neutral	neutral	Upper Lt 5 Rt 5	Lt normal	1 red
			Lower Lt 5 Rt 5	Rt normal	0 yellow

Tx = Treatment; Wk = week; Lt = left; Rt = right; LLD = leg length discrepancy; ROM = range of motion; S = shoulder; P = pelvis.

**Table 2:** Visual Analogue Scale pain ratings *.

	Leg	Hip	Knee	Ankle	Shoulder	Arm	Elbow	Wrist	Low Back
Before treatment began	10.0	4.25	8.25	8.25	4.75	4.75	3.75	5.5	7.5
Before 3rd treatment	4.75	3.25	5.75	3.25	2.0	1.75	5.5	2.5	2.5
Before 8th treatment	2.75	0.25	2.75	1.5	1.5	1.5	0.5	0.5	0.5
Percent reduction	72.5	94.1	66.7	81.8	68.4	68.4	86.7	90.9	93.3
Pain at 6-month follow-up **	5.0		5.0	6.0				3.0	1.0

* 0 means no pain at all; 10 means worst pain possible. ** patient did not rate all locations.

**Table 3:** Location and number of treatment pulses *.

Treatment	Skull	Cervical	Lumbar	Sacrum/Iliac	Other
1 Wk1-T		Lft C1 80			
2 Wk1-W	Cz40				
3 Wk1-Th			L5 ^†^ 60	Rt S1 60	
4 Wk1-F		Lft C1 80			
5 Wk2-Su		Lft C1 80			
6 Wk2-Th	Cz 40				
7 Wk2-F	Cz 40				
8 Wk2-Sa					Epigastrium 40
9 Wk3-W	Cz 40				
10 Wk3-Th		Lft C1 80			
11 Wk3-F	Cz 40		L5 ^†^ 60		
12 Wk4-F		Lft C1 80			
13 Wk5-M	Cz 40	Lft C1 80			
14 Wk5-T	Cz 40	Lft C1 80		S1 ^†^ 60	Abdomen RLQ 40
15 Wk5-W	Cz 40	Lft C1 80			
16 Wk5-Th				S3 ^†^ 60	
17 Wk5-F				Rt S4 60	
18 Wk7-M		Lft C1 80			
19 Wk8-T	Cz 40				Rt knee lateral 80
20 Wk8-W	Cz 40				
21 Wk8-F	Cz 40				

Wk = week; Lft = left; Rt = right; RLQ—right lower quadrant; ^†^ Midline. * Each pulse lasts about 3 s including the frequency reset.

**Table 4:** Comparison of past experience with six-month follow-up of sensory, vasomotor, sudomotor/edema, and motor/trophic conditions *.

Symptom	Past Experience	Present at 6-Month Follow-Up
Burning pain	4	2
Increased skin sensitivity to touch	4	3
Changes in skin temperature: warmer or cooler compared to the opposite extremity	4	1
Changes in skin color: often blotchy, purple, pale, or red	4	2
Changes in skin texture: shiny and thin, sometimes excessively sweaty	4	4
Changes in nail and hair growth patterns	4	4
Swelling and stiffness in affected joints	4	3
Motor disability, with decreased ability to move affected body parts	4	4

* Scale where 0 = not at all and 4 = all the time.

## Data Availability

All basic clinical data have been reported. Physiological assessment data for all treatments and initial cervical X-rays are available upon request from the corresponding author.
